# Feasibility of Baseline cardIac investiGation in awake elepHants (*Elephas mAximus*) using tRansThoracic echocardiography: The BIG HEART study

**DOI:** 10.1371/journal.pone.0333463

**Published:** 2026-02-02

**Authors:** Antonin Boutibou, Valérie Chetboul, Mathieu Magnin, Morgan Bureau, Coline Baillon, Norin Chai

**Affiliations:** 1 La Tanière - Zoo refuge, ferme du Grand, Nogent-le-Phaye, France; 2 École nationale vétérinaire d’Alfort, CHUV-AC, Maisons-Alfort, France; 3 Univ Paris Est Créteil, INSERM, IMRB, Créteil, France; 4 UR APCSe Agressions Pulmonaires et Circulatoires Dans le Sepsis, VetAgro Sup, Université de Lyon, Marcy l’Etoile, France; 5 Unité de Physiologie, Pharmacodynamie et Thérapeutique, VetAgro Sup, Université de Lyon, Marcy l’Etoile, France; 6 Ménagerie, le Zoo du Jardin des Plantes, Muséum National d’Histoire Naturelle, Paris, France; 7 Yaboumba, Paris, France; Victor Chang Cardiac Research Institute, AUSTRALIA

## Abstract

**Introduction:**

Cardiovascular diseases are common in elephants, but most cases are diagnosed only postmortem. Transthoracic echocardiography (TTE) is widely used in domestic and captive animals, although its application in elephants has not yet been established. The aim of this pilot study was to determine the feasibility of performing TTE in Asian elephants (*Elephas maximus*), to develop a standardized procedure to optimize image quality, and to assess measurement variability.

**Materials and methods:**

Preliminary trials were conducted in ten elephants, including seven free-ranging animals in Cambodia and three captive elephants in France. These led to a standardized TTE protocol based on cooperative training and positive reinforcement. Key refinements included the exclusive use of a left parasternal approach, soaking the thoracic skin with lukewarm water before application of coupling gel, and extension of the left forelimb over a training ball to optimize access to the acoustic window. Each examination included two left ventricular (LV) views: a long-axis view for M-mode measurements and a short-axis view for two-dimensional (2D) analysis. A total of 72 examinations were performed on 4 days on three elephants, with offline assessment of eight 2D and M-mode variables, including two indices of LV function (fractional shortening and fractional area change). A general linear model was used to determine within-day and between-day coefficients of variation.

**Results:**

All examinations were successfully completed without anesthesia or restraint. Within-day and between-day variability were low for all parameters (coefficients of variation = 0–3.4%), while interanimal variability was higher (5%−19.6%).

**Discussion:**

LV dimensions and systolic function can be assessed in awake Asian elephants with excellent repeatability and reproducibility.

**Conclusions:**

The present validated TTE procedure enables longitudinal cardiac monitoring in captive Asian elephants without anesthesia, providing a unique opportunity for the antemortem diagnosis of left-sided heart disease and facilitating future comparative and conservation studies in this endangered species.

## Introduction

Cardiovascular diseases are increasingly reported as an important cause of morbidity and mortality in elephants, affecting both Asian elephants (*Elephas maximus*), listed as Endangered, and African elephants (*Loxodonta africana* and *Loxodonta cyclotis*), listed as Vulnerable and Critically Endangered respectively, according to the IUCN Red List [1–3]. Various cardiovascular disorders have been identified in these species, some of which result in sudden death or the development of congestive heart failure. Documented noninfectious acquired conditions include dilated and hypertrophic cardiomyopathies, as well as arteriosclerotic changes primarily affecting the aorta, coronary arteries, and major aortic branches [1,2,4]. In addition, viral myocarditis cases associated with Picornaviridae or West Nile virus infections have also been described [1,4]. Several retrospective necropsy surveys have highlighted the importance of cardiovascular pathology in both Asian and African elephants [1,2]. In a survey of 379 captive elephants, cardiovascular disease was documented in only 5% of cases, but 95% of these animals died, with cardiac pathology directly responsible for 61% of the deaths [1]. In a more recent study analyzing 336 necropsy reports from the European Association of Zoos and Aquaria, cardiovascular diseases represented the leading category of lesions in African elephants (24% of cases) and the third most common category in Asian elephants (15% of cases), after infectious diseases (notably, elephant endotheliotropic herpesvirus, EEHV and musculoskeletal disorders [2]). Additionally, infection with EEHV, a major cause of mortality in young Asian elephants, targets endothelial cells and can induce severe vascular dysfunction and myocardial injury [5,6]. Such data underline both the clinical importance of cardiac disorders in elephants and the fact that most knowledge to date has been derived from necropsy rather than functional investigations in living animals.

In human and veterinary cardiology, in both domestic and captive animals, transthoracic echocardiography (TTE) is the gold standard non-invasive imaging modality for assessing cardiac anatomy and function, allowing real-time evaluation of chamber dimensions, wall thicknesses, myocardial contractility, and valve assessment, thereby facilitating early recognition of pathological alterations and guiding therapeutic interventions [7]. Despite its widespread application across numerous animal species, echocardiographic data remain extremely limited in elephants, and its clinical use is still rare. The paucity of such data can be attributed not only to the large body size but also to the particularly thick skin and subcutaneous tissues, and to the cranial and relatively deep position of the heart within the thorax, which limit acoustic access [1]. Additional logistical and ethical constraints also contribute to this scarcity, as echocardiographic evaluation requires close physical proximity to the thorax of these massive animals, ideally in standing, awake conditions to avoid the confounding effects and risks of anesthesia, but with inherent safety concerns for operators. To the best of our knowledge, only fragmented echocardiographic observations have been reported to date, with no standardized protocol or population-based reference values available to guide interpretation.

Given the prevalence and impact of cardiac diseases in elephants, establishing a repeatable, reproducible, and non-stressful TTE procedure is essential for preventive and diagnostic strategies. Reliable antemortem cardiac assessment tools, such as TTE, are therefore needed to detect such complications that are most often recognized only postmortem.

The aims of the present pilot study were therefore 1) to determine the feasibility of performing TTE in awake, apparently healthy Asian elephants (*Elephas maximus*), 2) to develop a standardized and non-stressful TTE procedure to optimize image quality, and 3) to assess the intra-observer within-day (repeatability) and between-day (reproducibility) variability of the corresponding measurements.

## Materials and methods

### Animals

Feasibility echocardiographic trials were conducted in ten Asian elephants (*Elephas maximus*), starting with seven free-ranging animals in Cambodia in collaboration with the Elephant Valley Project (the same cohort used by our group to develop an electrocardiogram (ECG) recording method in this species [8]).

Thanks to this experience and after confirming the feasibility of the procedure, we then refined and finalized the echocardiographic technique in three captive elephants from La Tanière – Zoo Refuge (Nogent-le-Phaye, France), which were the ones included in the validation protocol of the present study. These three elephants (EL1, EL2, and EL3) were males, apparently healthy as determined by routine veterinary monitoring, aged 7, 9, and 13 years, with shoulder heights of 2.02, 2.42, and 2.51 m, and body weights of 1,800, 2,600, and 3,600 kg, respectively.

The facilities housing the three study elephants (EL1, EL2, and EL3) included one indoor enclosure, with controlled access to two outdoor areas. The indoor and outdoor enclosures contained furnishings that added physical complexity to their environment (e.g., rocks, scratching poles, suspended food access). A staff of four keepers cared for the elephants, with at least two present on site each day. In addition, one full-time veterinarian was responsible for their health care and welfare. The elephants were fed twice daily with hay, forage, and browse. They also received daily enrichments (feeding, physical, and cognitive), using apples, bananas, mangos, and training biscuits (DK training biscuits L, DK Zoological, Putten, The Netherlands). The elephants also participated in twice-daily medical and husbandry training sessions based on positive-reinforcement operant conditioning, each lasting approximately 15–45 minutes per elephant. During these sessions, animals were progressively conditioned to voluntarily present the targeted body region and remain stationary while keepers performed specific procedures. Correct behaviors were systematically reinforced using a whistling sound as a secondary reinforcer, immediately followed by a food reward (e.g., a piece of fruit) and verbal praise. This training framework follows previously published approaches in cooperative care for captive elephants [9–12].

### Ethical considerations

The cardiac ultrasound examinations performed in this study were carried out without the use of anesthesia, as part of routine clinical care, on captive elephants regularly trained to voluntarily participate in veterinary procedures through positive reinforcement techniques. All elephants remained in their familiar environment throughout the whole study period. These animals undergo daily training sessions that enable them to accept medical procedures such as foot presentations for pedicures, oral inspections, and even blood sampling for preventive health assessments without the need for restraint or stress. Consequently, the echocardiographic examinations were fully integrated into an established health-monitoring program and did not involve any experimental design or intervention beyond standard clinical practice. They therefore cannot be considered animal experimentation or any additional intervention that could compromise animal welfare.

This type of cooperative training is now widely recognized as essential to the management of captive elephant health, serving both animal welfare and handler safety [9,10]. It avoids the use of invasive procedures, physical restraint, or anesthesia, while promoting voluntary cooperation during clinical interventions [11,12]. Moreover, such training is considered a form of cognitive and social enrichment, contributing positively to the animals’ emotional and physical well-being. Within this framework, the procedures described here fall strictly under the scope of routine veterinary practice, and thus did not require prior approval from an ethics committee according to experimental animal use regulations. They are also in line with international standards for the management of captive elephants, as emphasized by the Association of Zoos and Aquariums [12], which highlights voluntary training to minimize the need for anesthesia, and by the European Association of Zoos and Aquaria [13], which explicitly recommends cooperative training for diagnostic procedures such as ultrasound examinations.

### Transthoracic echocardiographic technique and measurements

#### Development and feasibility of the transthoracic echocardiographic procedure in 10 elephants (Figs 1 and 2).

**Fig 1:**
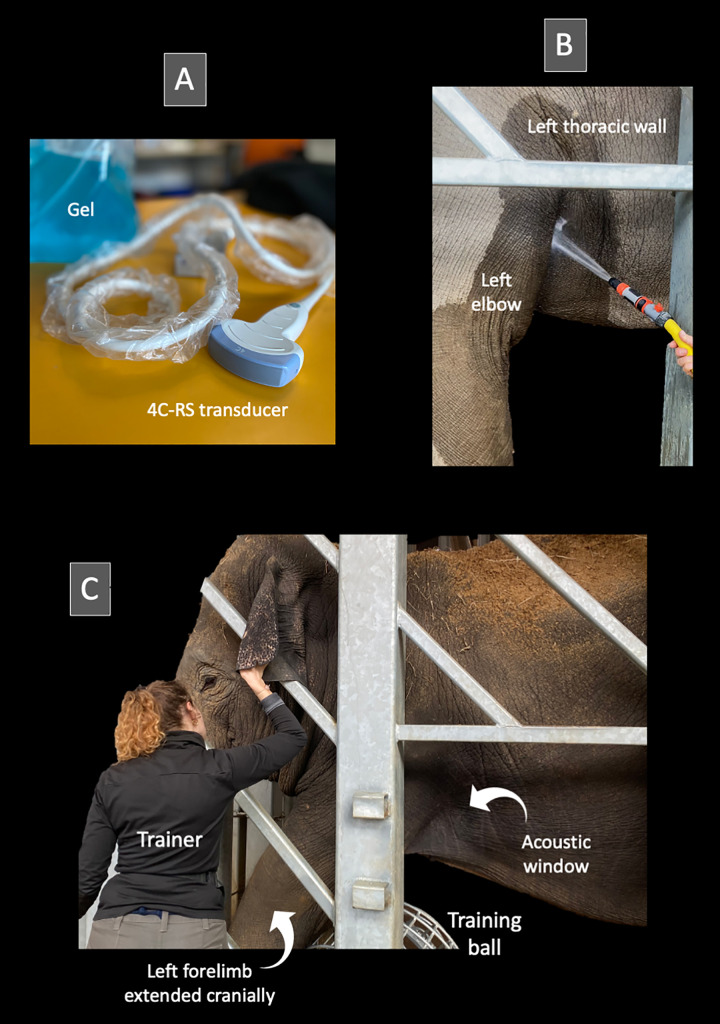
(A–C) Echocardiographic procedure in the elephants included in the study. **(A)** A 4C-RS convex transducer was used, which provided a wide ultrasound field suitable for cardiac imaging in this large species. **(B)** Prior to imaging, the acoustic window located slightly dorsal and caudal to the left elbow was rinsed with lukewarm water to soften the thick skin and improve acoustic coupling with the large amount of ultrasound gel applied, a step that was well tolerated and appeared to be positively perceived by the elephants. **(C)** The elephant stood calmly and without restraint against the protective barrier while the trainer gently interacted with its head. The left forelimb was extended cranially, facilitated by resting it over a training ball placed on the ground, in order to optimize access to the cranial portion of the left cranial thoracic wall and echocardiographic window. *Photos by Valérie Chetboul.*

All TTE examinations were performed using a portable cardiovascular ultrasound system (Vivid iq, GE Healthcare, 9900 Innovation Drive, Wauwatosa, WI 53226, USA) equipped with a 4C-RS convex transducer (1.8–6 MHz). Our preliminary trials demonstrated that the 4C-RS probe facilitated identification of the heart – a challenging step given the size of the animal – and provided a wider field of view, particularly in the cardiac long-axis plane, compared with the 3S phased-array transducer (1.5–3.5 MHz). The 4C-RS transducer was therefore selected for this study (Fig 1A). The main echocardiographic settings were as follows: a depth of 33 cm, a field of view adapted to include the entire left ventricle (LV) in long-axis and short-axis views, a transducer frequency of 1.5 MHz with tissue harmonic imaging at 3 MHz, and a frame rate of 13 frames per second.

**Fig 2:**
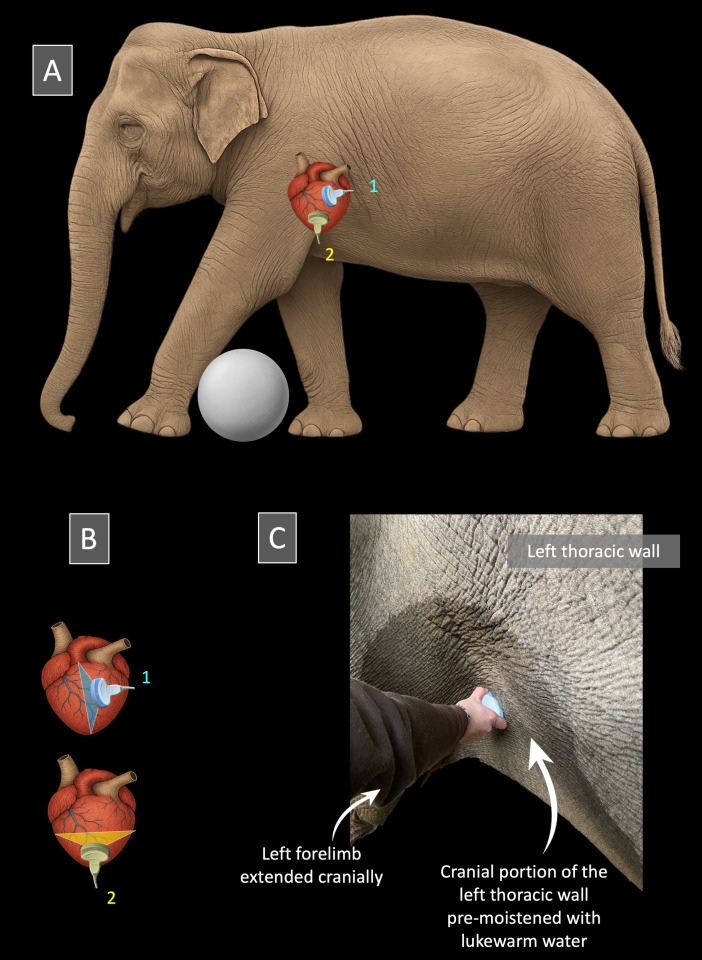
(A-C) Transducer positioning for echocardiographic imaging of the elephants included in the study. **(A) and (B)** Elephant positioning for transthoracic echocardiographic examination and probe placement to obtain the left parasternal long-axis view (1) and the left parasternal short-axis view at the level of the left ventricle (2). **(C)** Position of the transducer to obtain the left parasternal long-axis view. The probe is placed in an intercostal space located caudal and slightly dorsal to the left elbow, aligned parallel to the ribs. In both Fig 2A and 2C, the left forelimb is gently extended cranially, facilitated by resting over a training ball placed on the ground, in order to improve access to the cranial portion of the left cranial thoracic wall. For optimal imaging, this acoustic window was first moistened with lukewarm water to soften the skin, before application of a large amount of ultrasound gel, thereby enhancing ultrasound transmission. *Illustration and photo by Valérie Chetboul.*

Animal training for the TTE procedure was based on operant conditioning with exclusively positive reinforcement. The refinement of the technique initially consisted of identifying the optimal acoustic window through preliminary echocardiographic trials first conducted in seven free-ranging Asian elephants in Cambodia [8]. Using this initial experience, we then finalized the echocardiographic procedure in the three elephants (EL1, EL2, EL3) included in the present validation study. Attempts to obtain right parasternal views – which represent the standard imaging approach in domestic carnivores and horses [7] – proved unsuccessful, as the heart was either not visible or only poorly visualized depending on the individual. Consequently, only the left parasternal approach was retained, with the probe positioned on the left hemithorax, caudal and dorsal to the left elbow, in the presumed fourth intercostal space. Using this left parasternal approach, the LV could be consistently visualized in two-dimensional (2D) mode in both its long-axis and short-axis planes, hereafter referred to as the left parasternal long-axis view and the left parasternal short-axis view, respectively. In contrast, the right heart chambers, the aorta, and the left atrium were only poorly visualized with this approach and were therefore not measured.

Preliminary trials also revealed that image quality was improved by a two-step preparation of the acoustic window: first, the thick skin was soaked with lukewarm water, ensuring sufficient time for adequate softening (i.e., at least 3–5 minutes under water, followed by an additional 5 minutes of waiting), and then the application of a generous amount of coupling gel (Fig 1B).

#### Final transthoracic echocardiographic procedure used in the three elephants EL1, EL2, and EL3 (Figs 1 and 2).

The three elephants included in the validation study were trained to remain standing for up to 5 minutes against the enclosure. They were then gradually desensitized to the preparation of the acoustic window, which involved soaking the skin with lukewarm water and then applying generous amounts of ultrasound coupling gel to the thorax. Finally, they were accustomed to the placement of the probe on their thorax without reacting to the procedure. After three months of training, the three elephants were readily conditioned to voluntarily maintain the optimal position for up to 5 minutes, allowing acquisition of the TTE images.

The final TTE method was as follows: the trainers were responsible for bringing the elephants to an appropriate location, as close as possible to the protective barrier (Fig 1C). They also trained the elephants to move their left forelimb forward (using a large training ball as a target for positive reinforcement), in order to optimize access to the echocardiographic window located cranially, immediately caudal and slightly dorsal to the left elbow (Fig 1C). While the trainers distracted the elephants by gently talking to them and offering food (apples), optimal acoustic windows were promptly identified and obtained by one observer (Observer 1, AB), while a second observer (Observer 2, VC, Diplomate of the European College of Veterinary Internal Medicine – Cardiology) simultaneously adjusted the imaging settings. The overall gain was set to its maximum to optimize visualization of the LV myocardial walls. All measurements were subsequently performed offline by Observer 2. Each examination systematically included the two aforementioned 2D views, i.e., the left parasternal long-axis view and the left parasternal short-axis view. The left parasternal long-axis view was used to position the M-mode cursor and obtain the transventricular M-mode view, allowing measurement of LV walls and cavity dimensions in both diastole and systole. Conventional M-mode was used. Given the presence of two operators during image acquisition, beam alignment could be optimized in real time from the left parasternal long-axis view, making anatomic M-mode reconstruction unnecessary. The left parasternal short-axis view was used to calculate an index of radial systolic LV function, namely the LV fractional area change (LV FAC%).

#### Left parasternal long-axis view and corresponding M-mode measurements performed in the three elephants EL1, EL2, and EL3.

**Fig 3:**
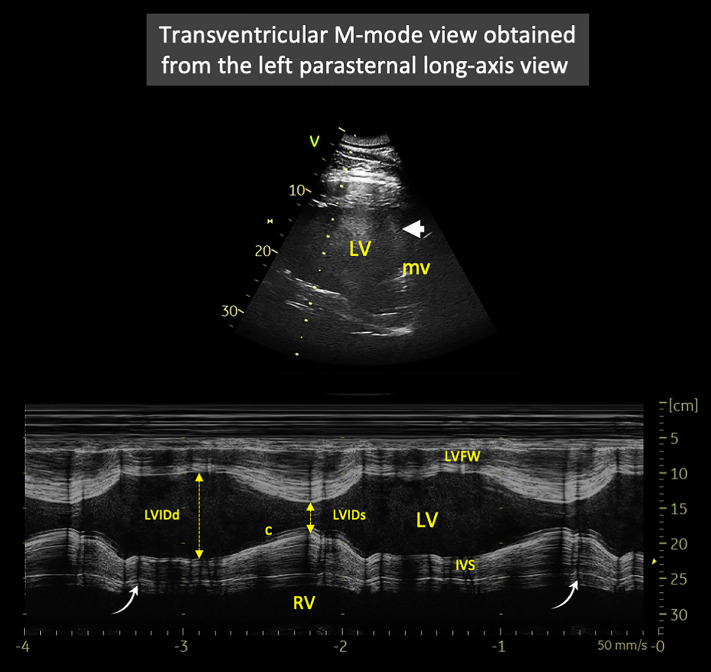
Representative transventricular M-mode echocardiogram obtained from the left parasternal long-axis view in one Asian elephant (*Elephas maximus*) from the study. This view shows two complete cardiac cycles (heart rate = 32 bpm), with the left ventricular free wall (LVFW) proximally and the interventricular septum (IVS) distally, delineating the left ventricular (LV) cavity. The M-mode cursor is positioned perpendicular to the LV myocardial walls, immediately below the insertion of the papillary muscles to exclude them from the measurements. Note the presence of chordae tendineae (c), which were not included in the diameter measurements. A spontaneous echo contrast is typically observed (arrowhead). In addition, vertical anechoic artifacts (curved arrows), corresponding to integumentary shadowing artifacts, are also seen (see text for explanation). *LVIDd and LVIDs: left ventricular internal diameter in end-diastole and end-systole, respectively. mv: mitral valve. RV: right ventricle. Image by Valérie Chetboul.*

The left parasternal long-axis view was obtained by placing the transducer in the intercostal space immediately caudal and slightly dorsal to the left elbow, with the ultrasound beam oriented parallel to the ribs and fine adjustments made according to image quality (Figs 2 and 3). This view displayed the LV cavity, bordered proximally by the LV free wall (LVFW) and distally by the interventricular septum (IVS). In some animals and examinations, a portion of the right ventricle was also visible in the lower part of the image (Fig 3). The M-mode cursor was positioned perpendicular to the LV myocardial walls, immediately below the insertion of the papillary muscles, which are particularly well developed in elephants, in order to avoid including them in the measurements. M-mode echocardiograms showing at least two consecutive cardiac cycles were obtained for measurement of LV diameters and myocardial wall thicknesses (Fig 3). Left ventricular end-diastolic and end-systolic internal diameters (LVIDd and LVIDs, respectively), LVFW thicknesses at end-diastole (LVFWd) and end-systole (LVFWs), IVS thicknesses at end-diastole (IVSd) and end-systole (IVSs) were measured on this view using the leading edge-to-leading edge technique, and the LV fractional shortening (FS%) was then calculated. As no concomitant ECG tracing could be performed, end-diastole and end-systole were defined based on mechanical events; specifically, end-diastole was identified as the moment showing the largest LV cavity, and end-systole as the moment showing the smallest LV cavity. As no arrhythmia was detected, heart rate (HR) was assessed by calculating the time interval between two consecutive maximal systolic excursions of the LV myocardial walls. Vertical anechoic artifacts (Fig 3) were frequently observed in all elephants and were likely related to their integumentary characteristics, including thick and irregular skin and coarse hair shafts, a feature not encountered in small carnivores. However, these artifacts did not interfere with the acquisition of measurements. Spontaneous echo contrast was also commonly observed, particularly in the left parasternal long-axis view (Fig 3).

#### Left parasternal short-axis view and corresponding two-dimensional measurements performed in the three elephants EL1, EL2, and EL3 (Figs 2 and 4).

**Fig 4:**
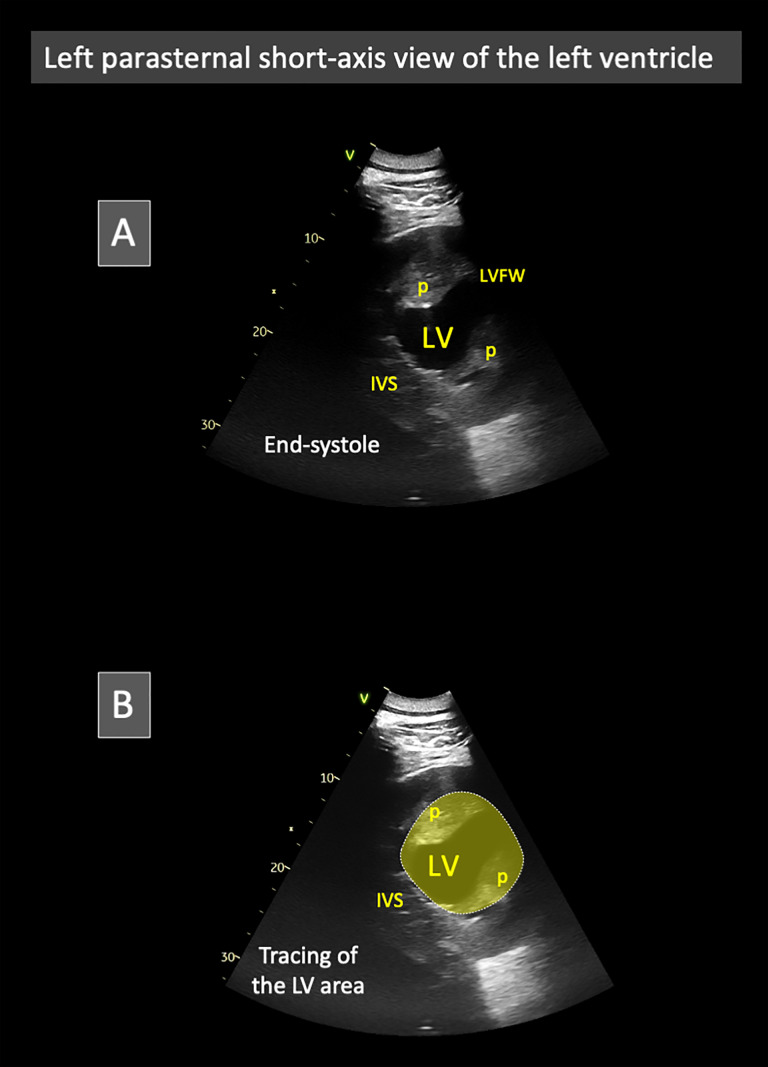
(A and B) Representative two-dimensional left parasternal short-axis view of the left ventricle in one Asian elephant (*Elephas maximus*) from the study at end-systole, showing the left ventricular (LV) cavity in cross-section and the two papillary muscles (p). An example of LV cavity tracing, excluding the papillary muscles, is illustrated in panel B. A similar tracing was performed on the frame obtained at end-diastole, and the LV fractional area change (FAC%) was calculated as [(LV area in diastole – LV area in systole)/ LV area in diastole] × 100. *IVS: interventricular septum. LVFW: left ventricular free wall. Image by Valérie Chetboul.*

The left parasternal short-axis view was obtained by keeping the transducer in the same intercostal space as for the long-axis view and by rotating it by approximately 70–90°, depending on the individual, resulting in a horizontal or nearly horizontal orientation (Fig 2). This view displayed the LV cavity in cross-section with its two papillary muscles (Fig 4), an orientation that differed from the conventional short-axis transventricular view typically obtained from the right parasternal approach in domestic carnivores and horses [7]. Using this view, the internal area of the LV was traced at end-diastole and end-systole (Fig 4), with the papillary muscles excluded from the tracing. The LV FAC% was calculated as the percentage change in LV cross-sectional area between diastole and systole [14]. As no concomitant ECG tracing could be performed, end-diastole and end-systole were defined based on mechanical events; specifically, end-diastole was identified as the frame showing the largest LV cavity, and end-systole as the frame showing the smallest LV cavity.

#### Assessment of intra-observer within-day and between-day variability of TTE variables in the three elephants EL1, EL2, and EL3.

A total of 72 echocardiographic examinations were performed on 4 different days over a 3-month period in the three elephants EL1, EL2, and EL3, using the above-described echocardiographic procedure. Each elephant underwent six echocardiographic examinations per day, integrated into their routine training sessions. Image acquisition for each examination lasted no more than 5 minutes, and the procedure was interspersed with the elephants’ usual training exercises. This approach ensured that the examinations remained fully embedded within the animals’ cooperative training schedule without modifying the elephants’ usual daily routines or management practices, thereby minimizing any potential stress or disruption. Each examination included one 2D short-axis view of the LV and one 2D-guided transventricular M-mode view, with offline assessment of eight TTE variables (LVIDd, LVIDs, LVFWd, LVFWs, IVSd, IVSs, FS%, and LV FAC%).

### Statistical analysis

All analyses were conducted using RStudio (Version 2023.09.1 + 494), with the packages lme4, broom.mixed, dplyr, tidyr, writexl, and ggplot2. For each echocardiographic parameter, descriptive statistics (mean, standard deviation (SD), minimum, and maximum) were calculated for the entire dataset as well as separately for each individual elephant. A general linear model was used to determine the within-day and between-day coefficients of variation (CV). To assess measurement variability, linear mixed-effects models were fitted for each parameter with animal, day, and the animal × day interaction specified as random effects. This allowed partitioning of the total variance into three components: within-day variability (repeatability), between-day variability (reproducibility), and interindividual variability. For each source of variability, the standard deviation was extracted from the model, and the corresponding CV was calculated by dividing the SD by the overall mean of the parameter. This approach provided a normalized estimate of the magnitude of each variance component.

Additionally, the influence of HR, body weight, and age on echocardiographic parameters was investigated using multivariable linear mixed-effects models. For each parameter, HR, body weight, and age were included as fixed effects, while animal identity was modeled as a random intercept to account for repeated measures within individuals. Model estimates, 95% confidence intervals (CI), and P-values were extracted for each predictor. A P-value < 0.05 was considered statistically significant.

## Results

All 72 TTE examinations were successfully performed in the three elephants (EL1, EL2, EL3), and all eight variables, including six M-mode measurements (LVIDd, LVIDs, LVFWd, LVFWs, IVSd, and IVSs) and two derived indices (FS% from M-mode and LV FAC% from 2D mode), were assessable offline, yielding a total of 576 data points for the study. The mean HR ± SD during TTE examinations was 40 ± 4 beats/min (range: 32–47).

Table 1 presents the values of the 576 repeated TTE measurements for the entire cohort and for each elephant individually, and the corresponding within-day and between-day SD and CV values are shown in Table 2.

**Table 1:** Mean ± standard deviation (SD), minimum (Min), and maximum (Max) values of eight echocardiographic variables obtained from 72 transthoracic examinations performed in three Asian elephants (*Elephas maximus*, EL1–EL3). Results are presented for all elephants combined and individually. The echocardiographic variables included seven repeated M-mode parameters, i.e., interventricular septal thicknesses at end-diastole and end-systole (IVSd, IVSs), left ventricular internal diameters at end-diastole and end-systole (LVIDd, LVIDs) with calculation of the fractional shortening (FS%), and left ventricular free wall thicknesses at end-diastole and end-systole (LVFWd, LVFWs), as well as one two-dimensional parameter, the left ventricular fractional area change (LV FAC%). Heart rate (HR) was also calculated.

Echocardiographic variables	All the elephants					EL1					EL2					EL3				
	Mean	SD	Median	Min	Max	Mean	SD	Median	Min	Max	Mean	SD	Median	Min	Max	Mean	SD	Median	Min	Max
***Heart rate (beats per minute)***	40	4	40	32	47	42	2	43	38	46	38	3	38	32	43	39	4	39	33	47
***Left ventricular diastolic dimensions***																				
*LVFWd (mm)*	26.8	1.4	26.2	24.3	29.9	25.9	0.9	24.3	24.3	28.1	26.0	0.7	26.2	25.2	27.1	28.4	0.8	28.1	27.1	29.9
*LVIDd (mm)*	131.0	3.1	130.9	126.2	135.6	128.8	2.2	128.6	126.2	134.6	134.5	0.9	134.6	132.8	135.6	129.6	2.2	128.6	127.1	132.8
*IVSd (mm)*	26.4	1.7	26.2	23.4	29.0	24.7	0.9	25.7	23.4	26.2	26.4	1.1	26.2	25.2	29.0	28.1	0.8	28.1	26.2	29.0
***Left ventricular systolic dimensions***																				
*LVFWs (mm)*	55.7	4.2	52.4	49.6	63.6	52.7	1.9	50.5	49.6	56.1	53.0	0.7	52.4	50.5	54.2	61.2	1.3	61.3	58.9	63.6
*LVIDs (mm)*	57.3	9.4	53.3	45.8	72.0	52.7	1.1	53.3	49.6	54.2	70.1	1.5	70.1	67.3	72.0	49.1	2.6	48.6	45.8	54.2
*IVSs (mm)*	54.8	4.8	53.3	47.7	63.6	51.0	1.5	52.4	47.7	53.4	51.9	1.1	53.3	49.6	53.3	61.3	1.2	61.3	58.0	63.6
***Indices of left ventricular radial systolic function***																				
*FS%*	56	6	59	46	64	59	1	59	58	61	48	1	48	46	50	62	2	63	59	64
*LV FAC%*	60	4	61	51	66	61	2	62	57	64	55	2	56	51	58	63	2	63	60	66

**Table 2:** Within-day, between-day, and interindividual variability, expressed as standard deviations (SD) and coefficients of variation (CV), for eight echocardiographic variables obtained from 72 transthoracic examinations in three Asian elephants (*Elephas maximus*). These variables included seven repeated M-mode parameters, i.e., interventricular septal thicknesses at end-diastole and end-systole (IVSd, IVSs), left ventricular free wall thicknesses at end-diastole and end-systole (LVFWd, LVFWs), and left ventricular internal diameters at end-diastole and end-systole (LVIDd, LVIDs) with calculation of the fractional shortening (FS%), as well as one two-dimensional parameter, the left ventricular fractional area change (LV FAC%).

Echocardiographic variables	Within-day variability		Between-day variability		Interindividual variability	
	SD	CV (%)	SD	CV (%)	SD	CV (%)
*Left ventricular diastolic dimensions*						
*LVFWd (mm)*	0.2	0.7	0.8	3.0	1.4	5.2
*LVIDd (mm)*	0.4	0.3	1.8	1.4	3.0	2.3
*IVSd (mm)*	0.3	1.2	0.9	3.4	1.6	6.2
*Left ventricular systolic dimensions*						
*LVFWs (mm)*	0.5	0.8	1.3	2.4	4.8	8.7
*LVIDs (mm)*	0.9	1.6	1.7	2.9	11.2	19.6
*IVSs (mm)*	0.0	0.0	1.3	2.4	5.7	10.3
*Indices of left ventricular radial systolic function*						
*FS%*	0.5	1.0	1.2	2.1	7.5	13.4
*LV FAC%*	0.0	0.0	1.9	3.1	4.3	7.2

All within-day and between-day CV values (n = 16) were < 10% (0 to 3.4%), the lowest being respectively observed for IVSs and LV FAC (0%), and LVIDd (1.4%). Interindividual variability was higher, ranging from 2.3% to 19.6%.

The effect of HR, body weight, and age on echocardiographic parameters was assessed using a multivariable linear mixed-effects model. Age was negatively associated with LVFWd (Estimate = –2.38 [95% CI: –4.64 to –0.12], P = 0.04), LVFWs (–9.46 [–18.37 to –0.55], P = 0.04), and LVIDd (–15.54 [–27.58 to –3.51], P = 0.01), and positively associated with LV FAC% (0.40 [0.40 to 0.50], P< 0.0001). Body weight was positively associated with LVIDd (0.80 [0.23 to 1.37], P = 0.01) and negatively with LV FAC% (–0.02 [–0.02 to –0.01], P < 0.0001). Lastly, HR was not significantly associated with any parameter in the multivariable models. Full results are presented in Table 3.

**Table 3:** Associations of heart rate (HR), body weight, and age with echocardiographic variables in Asian elephants (Elephas maximus) included in the study. HR are expressed in beats per minute, body weight in kilograms, and age in years. **Echocardiographic dimensions are in millimeters (mm), and indices in percent (%). Regression estimates are expressed per unit increase in the corresponding predictor. Multivariable linear mixed-effects models were fitted for each echocardiographic parameter with HR, body weight, and age as fixed effects and individual animal as a random effect. The table reports the estimated effect size (Estimate), the 95% confidence interval (CI), and the corresponding P-value for each predictor. Significant associations (P < 0.05) are shown in bold.** *IVSd and IVSs: interventricular septal thickness at end-diastole and end-systole, respectively. LVFWd and LVFWs: left ventricular free wall thickness at end-diastole and end-systole, respectively. LVIDd and LVIDs: left ventricular internal diameter at end-diastole and end-systole, respectively. FS%: fractional shortening. LV FAC%: left ventricular fractional area change.*

Parameter	Predictor	Estimate	Lower 95% CI	Upper 95% CI	P-value
***LVFWd***	HR	1.67	−3.78	7.12	0.54
	Body weight	0.06	−0.04	0.17	0.24
	**Age**	**−2.38**	**−4.64**	**−0.12**	**0.04**
***LVIDd***	HR	4.67	−24.36	33.71	0.75
	**Body weight**	**0.80**	**0.23**	**1.37**	**0.01**
	**Age**	**−15.54**	**−27.58**	**−3.51**	**0.01**
***IVSd***	HR	−3.12	−8.84	2.60	0.28
	Body weight	0.02	−0.09	0.14	0.67
	Age	−1.58	−3.95	0.79	0.19
***LVFWs***	HR	0.25	−0.82	1.32	0.64
	Body weight	0.46	−0.45	1.37	0.19
	**Age**	**−9.46**	**−18.37**	**−0.55**	**0.04**
***LVIDs***	HR	−2.62	−15.02	9.79	0.68
	Body weight	0.03	−0.21	0.27	0.81
	Age	−1.39	−6.53	3.75	0.59
***IVSs***	HR	4.87	−1.70	11.44	0.14
	Body weight	0.05	−0.08	0.18	0.46
	Age	0.77	−1.95	3.49	0.57
***FS%***	HR	−0.07	−0.17	0.02	0.14
	Body weight	0.02	−0.07	0.10	0.57
	Age	−0.34	−1.16	0.48	0.40
***LV FAC%***	HR	−0.08	−0.23	0.06	0.25
	**Body weight**	**−0.02**	**−0.02**	**−0.01**	**<0.0001**
	**Age**	**0.40**	**0.40**	**0.50**	**<0.0001**

## Discussion

Although echocardiography is considered the gold standard non-invasive imaging modality for the assessment of cardiac structure and function in humans and many animal species, its application in elephants has been extremely limited. To date, knowledge of elephant cardiology has mainly derived from necropsy and histopathological examinations [1,2], with only a few ante mortem investigations focusing on HR monitoring using ECG or wearable sensors [15–17], on specific ECG methodologies [8,18], or on circulating cardiac biomarkers such as cardiac troponin I in the context of EEHV infection [6,19]. These studies have provided valuable insights into normal cardiac rhythm and ECG tracings, stress-related HR changes, and myocardial injury, but they remain scarce and are most often limited by small sample sizes. To the best of the authors’ knowledge, no study has yet provided standardized, quantitative 2D or M-mode TTE data on cardiac morphology and function in awake elephants, and the antemortem diagnosis of heart diseases in this species thus remains challenging. This pilot study represents a critical step toward establishing a reliable diagnostic tool for the antemortem evaluation and longitudinal monitoring of left-sided cardiac disease in Asian elephants. For the first time, we demonstrate the feasibility of a standardized, repeatable, and reproducible TTE protocol in this species. Performing TTE in awake elephants opens new avenues for veterinary care, including pre-anesthetic evaluation, routine monitoring, early detection of subclinical cardiac disease, longitudinal assessment of disease progression, and evaluation of therapeutic interventions, thereby ultimately supporting the conservation of this threatened species. Additionally, integrating TTE monitoring into the management of Asian elephants at risk for, or affected by, EEHV could yield crucial insights into cardiac involvement and complement cardiac troponin I measurement in the overall clinical evaluation of this viral disease [6,19].

Until now, the limited development of TTE in elephants has primarily reflected the substantial technical challenges inherent to this species. Their massive body size complicates the rapid identification of acoustic windows, while the depth and large size of the heart, combined with its relatively cranial position beneath the forelimbs, restrict acoustic accessibility and permit only partial visualization of the organ with currently available equipment [1]. In addition, the large body size of elephants inevitably increases the imaging depth required to visualize the heart, which further contributes to signal attenuation and the need for optimized gain settings. Moreover, the unusually thick and relatively dry skin, together with abundant subcutaneous tissue, further attenuates the ultrasound beam. In addition to these anatomical and technical limitations, the behavioral challenges and potential risks for operators when handling awake elephants have further constrained the use of echocardiography in this species [1]. Collectively, these factors have long hindered the advancement of TTE in elephants. In the present study, several of these obstacles were successfully addressed through specific methodological refinements. The exclusive use of a left parasternal approach proved critical, as right parasternal imaging consistently failed to provide interpretable views owing to the thoracic conformation of the species. Careful preparation of the acoustic window (i.e., soaking the thick skin with lukewarm water, allowing sufficient time for softening, and then applying abundant ultrasound coupling gel) was also essential to ensure adequate image quality. The use of a convex 4C-RS probe further expanded the ultrasonic field compared with a phased-array transducer, while its relatively broad footprint fitted well within the wide intercostal spaces of elephants. In addition, optimized gain settings were required to compensate for both the considerable thoracic depth of the heart and the attenuation caused by thick skin and subcutaneous tissue. Extending the left forelimb over a training ball also improved access to the cranial portion of the left thoracic wall, thereby facilitating the acquisition of both long-axis and short-axis views of the LV. Collectively, these methodological steps, although simple, represent key innovations that enabled us to overcome the technical challenges inherent to TTE in elephants. Furthermore, as shown in Fig 4, it was not possible to achieve optimal alignment of the M-mode sampling line (i.e., perpendicular to both the IVS and the LVFW) using the left parasternal short-axis view. For this reason, the left parasternal long-axis view was used to obtain the M-mode echocardiograms. Conversely, the left parasternal short-axis view allowed complete visualization of the LV chamber in transverse section, which is the optimal view for assessing LV FAC% [14].

Spontaneous echo contrast was commonly observed in the LV chamber of the three elephants included in the study. This finding was likely related to the combination of low HR and the large size of the LV cavity, both of which promote low-flow conditions, further accentuated by the relatively high gain settings required for deep thoracic imaging.

Importantly, in this study the TTE protocol was developed through cooperative training and positive reinforcement, allowing elephants to participate voluntarily and remain calm throughout the procedure. This strategy avoided the need for sedation or physical restraint, both of which carry substantial risks in this species and are incompatible with repeated follow-up examinations. By relying on voluntary participation, the protocol is consistent with international animal welfare and safety guidelines [12,13] and illustrates how advanced diagnostic procedures can be incorporated into routine management without compromising the animals’ well-being. The absence of stress during the procedure was further supported by HR values recorded during examinations (mean = 40; range 32–47 beats/min), which remained within or near the upper end of values reported for non-stressed Asian elephants [17], even though the elephants examined here (7, 9, and 13 years old) were sub-adults, an age class in which slightly higher resting HR have been reported compared to fully mature adults. Nevertheless, HR variability was not assessed in the present study, and its evaluation would be of particular interest, as it is considered a more sensitive indicator of emotional state than HR alone [15].

The within-day variability of the TTE measurements was minimal, with very low SD and CV across all parameters (CV range: 0.0 to 1.6%), as was the between-day variability (CV range: 1.4 to 3.4%), together indicating high repeatability and reproducibility of both 2D and M-mode measurements (well below the 10% threshold) and demonstrating strong measurement consistency across sessions. By contrast, interindividual variability was more pronounced, with CVs ranging from 2.3% (LVIDd) to 19.6% (LVIDs), reflecting a substantial degree of physiological variation between elephants. Although the animals involved in the study were classified as sub-adults [17], they were at different stages of growth, as indicated by their distinct body weights (1,800–3,600 kg) and shoulder heights (2.02 to 2.51 m). This morphological heterogeneity likely accounts for much of the observed interindividual variation.

In the present study, several associations were observed between body weight or age, and TTE parameters using a multivariable linear mixed-effects model. As example, body weight was positively associated with LVIDd, which is consistent with the well-established scaling effect of body size on cardiac dimensions in other species [20–23]. As example, in horses, cardiac measurements are frequently indexed to body size using allometric scaling, which reduces interindividual variability and improves the biological interpretation of echocardiographic data [22,23]. Similar approaches may prove highly relevant in elephants once larger datasets become available, as suggested by equine studies demonstrating the value of such scaling methods [22,23]. Conversely, body weight was negatively associated with LV FAC%, suggesting that heavier elephants may have relatively lower systolic indices. Nevertheless, these findings must be interpreted with caution given the small sample size, but they highlight the importance of considering morphometric variables when interpreting echocardiographic data in elephants.

This preliminary work on TTE in Asian elephants presents several limitations. Quantitative measurements were restricted to the LV, as this chamber was the most consistently detectable and analyzable from the left parasternal approach. The left atrium, aorta, and right heart chambers could only be partially visualized and were therefore not assessed. Although a large number of repeated examinations (72) and measurements (576) were performed, the small number of animals included represents another limitation of the present study and restricts the robustness and generalizability of any associations identified between echocardiographic parameters and individual characteristics such as age, body weight, or HR. Furthermore, due to the prominent papillary muscles in the elephant LV, the positioning of the M-mode cursor in the left parasternal long-axis view differed from what is typically performed in small animals and horses [7]: it was placed more apically, at the level of papillary muscle insertion, to avoid their inclusion in the measurements (rather than just below the tips of the mitral leaflets at their maximal diastolic opening). Another limitation is the lack of simultaneous ECG recording, which precluded a precise temporal definition of end-diastole and end-systole. Although our group previously developed and validated a method for recording ECGs in elephants [8], combining ECG acquisition with TTE during the same session would have required additional restraint, equipment, and manipulation, thereby increasing the duration and complexity of the examinations and potentially inducing unnecessary stress in the animals. Because the primary objective of the present study was to assess the feasibility of performing TTE under voluntary, cooperative conditions, we chose to avoid adding further procedures that could compromise animal welfare or the quality of the echocardiographic data. Lastly, as all elephants included in this study were cooperative and accustomed to handling, the results presented here may not be directly applicable to non-trained or wild individuals. Extending this protocol to wild or untrained elephants will inevitably require adaptations, particularly because cooperative training cannot be implemented in these contexts. Moreover, as previously demonstrated by our group in dogs and cats [24,25], TTE is a highly observer-dependent examination. Therefore, the present results are valid only for the two observers involved (Observer 1 and Observer 2). The authors thus encourage veterinarians to determine their own intra- and inter-observer variability before undertaking further echocardiographic studies in elephants. Nevertheless, several comparable studies in horses have reported low to very low intra- and inter-observer variability for key M-mode and 2D measurements, confirming that echocardiography in large animals can achieve excellent repeatability and reproducibility [26,27]. Consistent with our results in elephants, equine studies have also shown that the largest source of variability for principal M-mode parameters (e.g., LVIDd, LVIDs) is the biological variation between individuals, which can be as high as nearly 80% [28].

## Conclusions

In conclusion, this study demonstrates that TTE can be successfully performed in awake captive Asian elephants following a relatively short period of cooperative training. The protocol established here enabled the assessment of eight 2D and M-mode variables with excellent repeatability and reproducibility, while requiring only minimal restraint. Future studies on larger populations of Asian elephants are now warranted to establish reference intervals for these TTE parameters, which will be essential for detecting pathological changes in chamber size and wall thickness, as well as for the diagnosis of LV dysfunction. This work lays the foundation for establishing TTE as a non-invasive and non-stressful diagnostic tool in elephants, representing a critical step toward integrating it into routine preventive health care and improving both clinical care and the long-term conservation of this threatened species.

## Supporting information

S1 File Raw echocardiographic data used for statistical analyses. This file contains the raw measurements obtained from the 72 transthoracic echocardiographic examinations performed in the three Asian elephants included in the study.
(XLSX)

## References

[1] Fowler ME, Mikota SK, editors. Biology, medicine, and surgery of elephants. Ames (IA): Blackwell Publishing; 2006.

[2] Hess A. Lesions found in the post-mortem reports of the Asian (Elephas maximus) and African (Loxodonta africana) elephants of the European Association of Zoos and Aquaria [Doctoral thesis]. Budapest (Hungary): University of Veterinary Medicine Budapest; 2021.

[3] IUCN. The IUCN red list of threatened species. Version 2021-3; 2021. Available from: https://www.iucnredlist.org

[4] Mikota SK, Sargent EL, Ranglack GS. The cardiovascular system. In: Mikota SK, Sargent EL, Ranglack GS, editors. Medical management of the elephant. West Bloomfield (MI): Indira Publishing House; 1994. p. 107–18.

[5] Simpson CF, Lewis AL, Gaskin JM. Encephalomyocarditis virus infection of captive elephants. J Am Vet Med Assoc. 1977;171(9):902–5. doi: 10.2460/javma.1977.171.09.902 200596

[6] Anderson KB, Steeil JC, Latimer E, Hall V, Hayek L-AC, Brandão J. Changes in serum cardiac troponin i in asian elephants (Elephas maximus) with elephant endotheliotropic herpesvirus infection. J Zoo Wildl Med. 2022;53(2):249–58. doi: 10.1638/2021-0003 35758566

[7] Boon JA. Evaluation of size, function, and hemodynamics. In: Boon JA, editor. Manual of veterinary echocardiography. 2nd ed. Ames: Wiley-Blackwell; 2011. p. 153–266.

[8] Chai N, Pouchelon JL, Bouvard J, Sillero LC, Huynh M, Segalini V, et al. Proposed simple method for electrocardiogram recording in free-ranging asian elephants (Elephas maximus). J Zoo Wildl Med. 2016;47(1):6–11. doi: 10.1638/2015-0162.1 27010258

[9] Fagen A, Mishra S, Wielebnowski N. Positive reinforcement training for medical procedures in captive Asian elephants (Elephas maximus): a case study of trunk wash training. J Appl Anim Welf Sci. 2014;17(3):250–60. doi: 10.1080/10888705.2014.912884

[10] Laule G. Positive reinforcement training and environmental enrichment: enhancing animal care, management, and welfare. J Exp Anim Sci. 2003;43(4):300–8. 10.2460/javma.2003.223.969 14552483

[11] Gurusamy N, Shanmugam K, Gopalakrishnan M, Chandrasekaran S, Sripathi V. A survey on elephant handling practices in India: implications for welfare and management. J Appl Anim Res. 2014;42(2):174–9. doi: 10.1080/09712119.2013.875918

[12] Association of Zoos and Aquariums (AZA). Elephant Care Manual. Silver Spring (MD): AZA Elephant TAG/SSP; 2012. Available from: https://assets.speakcdn.com/assets/2332/aza_standards_for_elephant_management_and_care.pdf

[13] European Association of Zoos and Aquaria (EAZA). Welfare standards for the accommodation and care of animals in zoos and aquaria. Amsterdam: EAZA; 2024. Available from: https://strapi.eaza.net/uploads/2024_04_WAM_Standards_Approved_06a0df2fe8.pdf

[14] Bonagura JD, Visser LC. Echocardiographic assessment of dilated cardiomyopathy in dogs. J Vet Cardiol. 2022;40:15–50. doi: 10.1016/j.jvc.2021.08.004 34750089

[15] Vézina-Audette R, Herry C, Burns P, Frasch M, Chave E, Theoret C. Heart rate variability in relation to stress in the Asian elephant (Elephas maximus). Can Vet J. 2016;57(3):289–92. 26933266 PMC4751769

[16] Kido N, Tanaka S, Omiya T, Kamite Y, Sawada K, Komatsu Y, et al. Emotion estimation using a wearable heart rate monitoring device in Asian elephants (Elephas maximus) during veterinary clinical procedures. J Vet Med Sci. 2020;82(6):856–60. doi: 10.1292/jvms.19-0637 32307341 PMC7324812

[17] Zhou F, Bao M, Guo X, Shen Q, Chen J, Li D, et al. Heart rate patterns of captive Asian elephant (Elephas maximus) in their natural habitat at Wild Elephant Valley, Xishuangbanna of China. Heliyon. 2024;10(4):e25720. doi: 10.1016/j.heliyon.2024.e25720 38370206 PMC10867636

[18] Bartlett SL, Abou-Madi N, Kraus MS, Wiedner EB, Starkey SR, Kollias GV. Electrocardiography of the Asian elephant (Elephas maximus). J Zoo Wildl Med. 2009;40(3):466–73. doi: 10.1638/2008-0140.1 19746861

[19] Gallini SH, DiGeronimo PM, Ward E, Thepapichaikul W, Tachampa K, Girolamo ND, et al. Evaluation of plasma cardiac troponin I in Asian elephants (Elephas maximus) using two clinical analyzers. J Zoo Wildl Med. 2023;53(4):654–60. doi: 10.1638/2021-0164 36640066

[20] Cornell CC, Kittleson MD, Torre PD, Häggström J, Lombard CW, Pedersen HD, et al. Allometric scaling of M-mode cardiac measurements in normal adult dogs. J Vet Int Med. 2004;18(3):311. doi: 10.1892/0891-6640(2004)18<311:asomcm>2.0.co;2 15188817

[21] Häggström J, Andersson ÅO, Falk T, Nilsfors L, OIsson U, Kresken JG, et al. Effect of body weight on echocardiographic measurements in 19,866 pure-bred cats with or without heart disease. J Vet Intern Med. 2016;30(5):1601–11. doi: 10.1111/jvim.14569 27573384 PMC5032876

[22] Huesler IM, Mitchell KJ, Schwarzwald CC. Echocardiographic assessment of left atrial size and function in warmblood horses: reference intervals, allometric scaling, and agreement of different echocardiographic variables. J Vet Intern Med. 2016;30(4):1241–52. doi: 10.1111/jvim.14368 27362277 PMC5108425

[23] Berthoud D, Schwarzwald CC. Echocardiographic assessment of left ventricular size and systolic function in Warmblood horses using linear measurements, area-based indices, and volume estimates: a retrospective database analysis. J Vet Intern Med. 2021;35(1):504–20. doi: 10.1111/jvim.15968 33247461 PMC7848374

[24] Chetboul V, Concordet D, Pouchelon JL, Athanassiadis N, Muller C, Benigni L, et al. Effects of inter- and intra-observer variability on echocardiographic measurements in awake cats. J Vet Med A Physiol Pathol Clin Med. 2003;50(6):326–31. doi: 10.1046/j.1439-0442.2003.00546.x 12887627

[25] Chetboul V, Athanassiadis N, Concordet D, Nicolle A, Tessier D, Castagnet M, et al. Observer-dependent variability of quantitative clinical endpoints: the example of canine echocardiography. J Vet Pharmacol Ther. 2004;27(1):49–56. doi: 10.1046/j.0140-7783.2003.00543.x 14995967

[26] Sampson SN, Jacobson RL, Sande RD, Susumi CJ, Larntz KJ, Tucker RL, et al. Reproducibility and repeatability of M-mode echocardiographic measurements collected from 25 normal horses. J Equine Vet Sci. 1999;19(1):51–7. doi: 10.1016/s0737-0806(99)80032-x

[27] Slack J, Durandot MM, Belcher CN, Collins N, Palmer L, Ousey J, et al. Intraoperator, intraobserver and interoperator variability of echocardiographic measurements in healthy foals. Equine Vet J Suppl. 2012;(41):69–75. doi: 10.1111/j.2042-3306.2011.00503.x 22594030

[28] Buhl R, Ersbøll AK, Eriksen L, Koch J. Sources and magnitude of variation of echocardiographic measurements in normal standardbred horses. Vet Radiol Ultrasound. 2004;45(6):505–12. doi: 10.1111/j.1740-8261.2004.04086.x 15605839

